# Case report: A novel *JAK3* homozygous variant in a patient with severe combined immunodeficiency and persistent COVID-19

**DOI:** 10.3389/fimmu.2024.1472957

**Published:** 2024-11-13

**Authors:** Renan Cesar Sbruzzi, Mayara Jorgens Prado, Bibiana Fam, Helena Ashton Prolla, Alessandra Hellwig, Grazielle Motta Rodrigues, Fernanda de-Paris, Mariana Jobim, Osvaldo Artigalás, Yoann Seeleuthner, Jean-Laurent Casanova, Jacinta Bustamante, Fernanda Sales Luiz Vianna

**Affiliations:** ^1^ Laboratory of Genomic Medicine, Center of Experimental Research, Hospital de Clínicas de Porto Alegre (HCPA), Porto Alegre, Rio Grande do Sul, Brazil; ^2^ Graduate program in Molecular and Cellular Biology, Department of Genetics and Molecular Biology – Federal University of Rio Grande do Sul, Porto Alegre, Rio Grande do Sul, Brazil; ^3^ Faculty of Medicine, Federal University of Rio Grande do Sul, Porto Alegre, Rio Grande do Sul, Brazil; ^4^ Laboratory Diagnostic Service, Hospital de Clínicas de Porto Alegre (HCPA), Porto Alegre, Rio Grande do Sul, Brazil; ^5^ Transplant Immunology and Personalized Medicine Unit, Hospital de Clínicas de Porto Alegre (HCPA), Porto Alegre, Rio Grande do Sul, Brazil; ^6^ Medical Genetics Service, Hospital de Clínicas de Porto Alegre (HCPA), Porto Alegre, Rio Grande do Sul, Brazil; ^7^ Genomic Medicine Program, Hospital de Clínicas de Porto Alegre (HCPA), Porto Alegre, Rio Grande do Sul, Brazil; ^8^ Laboratory of Human Genetics of Infectious Diseases, Necker Branch, INSERM U1163, Necker Hospital for Sick Children, Paris, France; ^9^ St. Giles Laboratory of Human Genetics of Infectious Diseases, Rockefeller Branch, The Rockefeller University, New York, NY, United States; ^10^ Department of Pediatrics, Necker Hospital for Sick Children, Paris, France; ^11^ Howard Hughes Medical Institute, The Rockefeller University, New York, NY, United States; ^12^ Study Center for Primary Immunodeficiencies, Necker Hospital for Sick Children, Assistance Publique-Hôpitaux de Paris Assistance Publique – Hopitaux de Paris (AP-HP), Paris, France; ^13^ Laboratory Research Unit, Hospital de Clínicas de Porto Alegre (HCPA), Porto Alegre, Rio Grande do Sul, Brazil; ^14^ National Institute of Population Medical Genetics (INAGEMP), Porto Alegre, Rio Grande do Sul, Brazil; ^15^ Graduate Program in Medicine: Medical Sciences, Universidade Federal do Rio Grande do Sul, Porto Alegre, Rio Grande do Sul, Brazil

**Keywords:** SCID, case report, *JAK3*, whole exome sequencing, prolonged SARS-CoV-2, inborn errors of immunity

## Abstract

Inborn errors of immunity (IEI) encompass a broad range of disorders with heterogeneous clinical presentations, often leading to challenges in early diagnosis. This study presents a case of a Brazilian patient with a T-B+NK- severe combined immunodeficiency (SCID) diagnosed at the age of 6 months when was admitted to the hospital due to multiple infectious diseases. Despite undergoing hematopoietic stem cell transplantation (HSCT), the patient had recurrent infections, requiring constant hospital care, including IgG infusions and several antibiotic treatments for the following months. One year after HSCT, presenting mixed chimerism, the patient tested positive for SARS-CoV-2 in nasopharyngeal, duodenum, and intestine samples, with persistent positive tests over a six-month period. Whole exome sequencing identified a private homozygous missense variant (c.1202T>C; p.Leu401Pro) in the Janus Kinase 3 (*JAK3*) gene. This substitution is located in a highly conserved position, and different bioinformatic variant effect predictors classified the variant as damaging. In silico structural analysis suggested that the variant led to increased structural instability, disrupting the hydrophobic interactions within the SH2 domain, thereby influencing the neighboring residues and potentially altering the interaction between *JAK3* and gamma chain (γc) intracellular receptors. This study provides evidence for the novel pathogenicity classification of the variant and highlights the importance of the *JAK3* and SH2 domain modulating protein function and their contribution to the SCID pathogenesis.

## Introduction

Severe combined immunodeficiencies (SCIDs) are a group of Inborn Errors of Immunity (IEI) characterized by abnormalities in development and function of the adaptive immune system. Patients with SCID present thymopoiesis defects that results in both numeric and functional impairment of T cells and, depending on the genetic variants responsible for the condition, defects in B and natural killer (NK) cells might also be found (1). The T-B+NK- phenotype SCID has been associated with cytokine signaling abnormalities, caused by either deficiency in the gamma chain (γc) subunit, encoded by the interleukin 2 receptor subunit gamma (*IL2RG*), or Janus Kinase 3 (*JAK3*) deficiency (1, 2). SCID patients generally present no symptoms on their first days of life, but soon they develop severe opportunistic infections and present higher susceptibility to attenuated-pathogen vaccine-associated infections (1). Unless proper treatment is early administered, usually immunoglobulin reposition and hematopoietic stem cell transplantation (HSCT), affected individuals have a significant risk of mortality within the first year of life (2, 3). SCID patients as well as patients with other IEIs, are susceptible to severe COVID-19 (4) and to persistent SARS-CoV-2 infection (5). The delayed viral clearance in those patients leads to prolonged periods of viral replication and chronic infection, increasing the risk of viral transmission and prompting medical care for longer periods (5, 6). In addition to patient management challenges, prolonged SARS-CoV-2 infection may also present an epidemiological risk, as the immune status of the host can create different selective pressures that may allow the emergence of new variants of concern (7). In this study, we report a patient with a T-B+NK- SCID associated with several pulmonary infections, BCG-itis and a six-months long SARS-CoV-2 infection, who presented a new homozygous missense variant in *JAK3* gene.

## Case presentation

**Figure 1:**
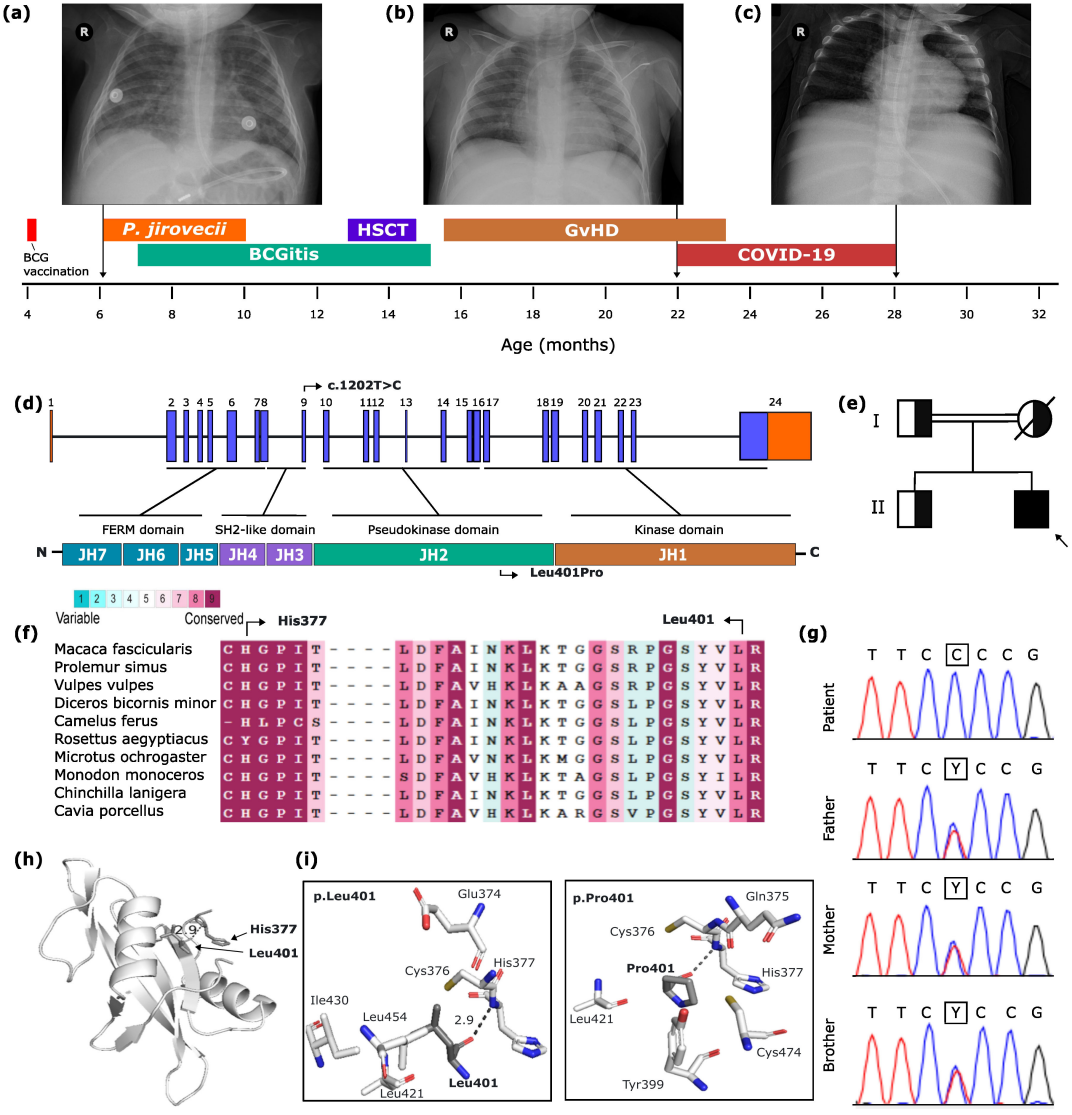
Brazilian patient with SCID due to *JAK3* deficiency. SCID case report findings, patient’s timeline describing relevance events, and related X-ray findings. In **(A)** Lung hyperinflation signs. Peribronchovascular bundles, especially in the central lung areas, along with faint heterogeneous opacities, primarily in the lower parts of the left lung and upper parts of the right lung. **(B)** Mild infiltration in the medullary region of the left lung, with no other evidence of pleuropulmonary alteration. **(C)** Mild thickening of bronchial walls and apparent splenomegaly can be seen. **(D)** *JAK3* gene and protein. *JAK3* gene comprises 24 exons (23 coding) that code seven Janus kinase homology domains (JH1-JH7) highlighting JH3 and JH4 together constitute the SH2-like domain, and JH4 domains constitute the FERM domain. **(E)** Family pedigree. **(F)** Multisequence alignment colored according to conservation scores, highlighting p.His377 and p.Leu401. **(G)** Electropherogram showing the variant’s segregation pattern on the kindred (Y= C+T). **(H)** 3D structure of *JAK3* SH2 domain. **(I)** 3D stick structure of residues under a distance of 4 angstroms of p.Leu401 (left) and p.Pro401 (right).

A 6-month-old Brazilian boy of a first-cousin consanguineous couple, with no gestational complications or history of immunodeficiency, was admitted at the hospital’s Intensive Care Unit (ICU) in March 2020, presenting sibilant breathing, severe respiratory dysfunction, and cyanosis (**Figures 1A–C**). As newborn screening for T and B cell deficiencies isn’t fully implemented in Brazil, the boy received all vaccines following the Brazilian National Calendar of Vaccination (8), including *Bacillus Calmette-Guérin* (BCG) at 4 months of age, which later caused BCG-itis. He was treated using rifampicin (18 mg/kg/day), isoniazid (14.5 mg/kg/day), and ethambutol (24 mg/kg/day) intermittently in the next following months. The chest X-Ray showed signs of bronchopneumopathy (**Figure 1A**) and *Pneumocystis jirovecii*, *Pneumococcus* and *Bordetella* were detected on bronchoalveolar aspirate. Pneumocystosis was treated with oral trimethoprim (TMP)- sulfamethoxazole (SMZ) (4 mg of TMP and 20 mg of SMZ/kg/day) for approximately two months, in addition of courses of fluconazole (6 mg/kg/day) and prednisolone (0.47 mg/kg/day) to manage and prevent other infections. *Pneumococcus* and *Bordetella* infections were managed with courses of amoxicillin (70 mg/kg/day) and azithromycin (10 mg/kg/day). First immunological screening found low immunoglobulin G (IgG <108 mg/dL, with reference values for this age around 203 mg/dL) and the absence of T cells (CD3/CD4 and CD4-/CD8-) as well as NK cells (CD53+/CD16+/CD3-), and low counts (0.1%) of T CD3 and CD3/CD8 cells. The patient also presented 99% of his lymphocyte population composed of CD19 and CD19/20 B cells. The immunophenotypic analysis allowed the classification of SCID as T-B+NK- phenotype and he was referred to HSCT. Molecular diagnosis for SCID was not performed at assistance care. Forty days after admission the patient received ICU discharge.

At 13 months old, conditioned HSCT was conducted using stem cells from his father (**Table 1**). The patient continued presenting hypogammaglobulinemia and was treated with periodic intravenous immunoglobulin infusions. One month after HSCT, the patient presented ulceration on BCG scar and fever, followed by erythematous skin rashes disseminated throughout the whole body. Soon after BCG-itis treatment, the patient was diagnosed with cutaneous manifestation of graft versus host disease (GvHD). As this was a case of steroid-refractory GvHD, the patient did not respond to treatment with methylprednisolone, basiliximab, needing the application of five mesenchymal cells infusion to halt disease progression (**Figure 1B**). One year after the HSCT, the patient had an ICU admission due to respiratory symptoms and radiological findings indicating extensive lung damage (**Figure 1C**). A PCR test was conducted and SARS-CoV-2 was detected. The patient suffered from an episode of bronchospasm, which was managed with noninvasive ventilation and salbutamol, and was discharged after 10 days. In the following months, the patient had constant hospital admissions related to both skin lesions due to GvHD and opportunistic infections, such as urinary tract infection by *Enterobacter kobei*, catheter-associated infection by *E. faecium, and infection by Clostridium difficile*. The patient was again tested for SARS-CoV-2 in November and December 2021, presenting persistently positive results and viable viral particles throughout this period, as well as increased viral load over time (9). In addition, he presented persistence of respiratory and gastrointestinal symptoms. SARS-CoV-2 was tested and detected in duodenum and intestine, but not in stomach samples. Six months after the first positive result for SARS-CoV-2 infection, the patient obtained his first negative test result for SARS-CoV-2 from nasopharyngeal samples. Currently, the patient is undergoing regular follow-up and, despite HSCT in 2020 and of the recovery of T and NK cell populations, he still presents mixed donor chimerism, close to 40% (**Table 2**).

**Table 1:** Summary of HLA typing results using different resolutions methods.

Individual and HLA Typing	Typing Results				
	A*	B*	C*	DRB1*	DQB1*
Patient					
HLA medium resolution	03:CCCFK, **32**:CCCFM	**15**:CCFNZ, 15:CCFPA	02:CEJNK, 03:CEJNN	**08**:CCNU, 13:CCTBH	NP
HLA high resolution	NP	NP	NP	**08**:04, 13:01	**04**:02, 06:03
Donor (patient’s father)					
HLA medium resolution	11:CDDTR, **32**:CEKZY	**15**:CCCKD, 35:CCKE	NP	01:CCVMC, **08**:CCENU	NP
HLA high resolution	NP	NP	NP	01:01, **08**:04	**04**:02, 05:01
HLA match	32	15	–	08	04
	HLA match rate: 4/8				

NP, not performed; HLA, human leukocyte antigen.

Bold values means the allele number matching between the donor and receptor.

The * symbol is a standard delimiter that separates the gene from the allele in the nomenclature used to describe HLA variants, as part of a formal naming system.

**Table 2:** Patient’s immunophenotyping findings post HSCT.

		Months post HSCT							
		1 month	4 months	8 months	12 months	16 months	21 months	25 months	30 months
**T** **cells**	Patient age (months)	14	18	21	26	29	34	38	43
	Lymphocytes	574 (3245-6981)	958 (3245-6981)	609 (3245-6981)	219 (2210-5804)	671 (2210-5804)	837 (2210-5804)	2096 (2210-5804)	3566 (2210-5804)
	CD3	514 (1907-4314)	791 (1907-4314)	594 (1907-4314)	218 (1498-3816)	653 (1498-3816)	807 (1498-3816)	1300 (1498-3816)	3003 (1498-3816)
	CD4	242 (957-2727)	71 (957-2727)	415 (957-2727)	163 (786-2086)	452 (786-2086)	547 (786-2086)	692 (786-2086)	1997 (786-2086)
	CD8	263 (563-1753)	70 (563-1753)	138 (563-1753)	41 (452-1701)	119 (452-1701)	214 (452-1701)	400 (452-1701)	849 (452-1701)
**T CD4+**	CD45RA+/CD27+ (Naïve)	1 (291-1636)	2 (291-1636)	76 (291-1636)	21 (287-1276)	16 (287-1276)	75 (287-1276)	259 (287-1276)	1472 (287-1276)
	CD45RA-/CD27+ (Central memory)	165 (124-434)	61 (124-434)	282 (124-434)	120 (136-436)	339 (136-436)	398 (136-436)	380 (136-436)	485 (136-436)
	CD45RA-/CD27- (Effector memory)	75 (60-335)	9 (60-335)	54 (60-335)	18 (86-263)	95 (86-263)	72 (86-263)	50 (86-263)	38 (86-263)
	CD45RA+/CD27- (Effector cells)	NA	NA	4 (94-741)	3 (74-405)	2 (74-405)	2 (74-405)	3 (74-405)	2 (74-405)
**T** **CD8+**	CD45RA+/CD27+ (Naïve)	NA	5 (101-566)	71 (101-566)	27 (71-538)	17 (71-538)	80 (71-538)	126 (71-538)	610 (71-538)
	CD45RA-/CD27+ (Central memory)	150 (12.5-47)	268 (12.5-47)	28 (12.5-47)	6 (7-47)	37 (7-47)	103 (7-47)	142 (7-47)	98 (7-47)
	CD45RA-/CD27- (Effector memory)	29 (76-565)	354 (76-565)	24 (76-565)	5 (77-564)	53 (77-564)	16 (77-564)	11 (77-564)	32 (77-564)
	CD45RA+/CD27- (Effector cells)	NA	78 (197-757)	11 (197-757)	1 (122-672)	6 (122-672)	7 (122-672)	14 (122-672)	32 (122-672)
Donor chimerism (%)		53	20	19	7	19	16	41	NA
IgG		496	714	399	384	511	376	491	NA

Cell counts are shown as absolute counts/µL. Immunophenotyping reference range for the Brazilian population (10) under parenthesis. NA, Not available.

## Genetic investigation

Molecular investigation was performed through whole exome sequencing (WES) using DNA isolated from the patient’ whole blood, taken before HSCT. WES revealed a high homozygosity rate (3.8%), consistent with the parental consanguinity. Homozygous nonsense, missense, indel or splice site variants were prioritized. We further filtered variants with global low frequency (MAF <0.01) in public databases, and with a combined annotation dependent depletion (CADD) score higher than the gene’s mutational significance cutoff (MSC) (11), particularly in *IL2RG* and *JAK3* genes which are involved in T-B+NK- SCID (1). A novel homozygous missense variant was identified in the *JAK3* (NM_000215.4) gene, c.1202T>C, leading to the p.Leu401Pro substitution (**Figure 1D**). This private variant is classified as a variant of unknown significance (VUS) by the American College of Medical Genetics (ACMG) criteria (12). Sanger sequencing confirmed the variant to be in homozygosity in the patient, and in heterozygosity in his parents and brother (**Figures 1E, G**). The variant obtained a damaging score across most of the tested prediction tools (including CADD, AlphaMissense, SIFT, PolyPhen2, MAPP, MutationTaster, and MutPred2). The p.Leu401 residue, as analyzed with the ConSurf server (13), is at a high conservation level across different species, suggesting the functional importance of the residue position (**Figure 1F**). The p.Leu401 residue is located within the Src homology 2 (SH2) domain, an essential region for the protein signalization through interaction with the γc intracellular receptors. Furthermore, this residue forms a hydrogen bridge with a highly conserved residue, p.His377, located at the terminus of the FERM domain (**Figures 1H, I**). In addition, the novel variant led to increased structural instability, evidenced by chance in free energy (ΔΔG 0.15), as analyzed with STRUM (14) and cause a disruption in the hydrophobic interactions within the SH2 domain, thereby influencing the neighboring residues (**Figures 1H, I**).

## Discussion

In this study we describe a six-months-old Brazilian patient carrying a private *JAK3* variant c.1202T>C, leading to the p.Leu401Pro substitution, in homozygosity causing T-B+NK- phenotype of SCID who suffered from a prolonged SARS-CoV-2 infection. Brazil is a continental dimension country with economic constraints, making newborn screening nationwide implementation challenging. Studies have demonstrated the newborn screening’s (NBS) feasibility and benefits of this strategy in Brazil (16, 17), and they were fundamental to drive the approval of this strategy in Brazil. However, NBS still remains in an implementation stage nationwide, though a few states are currently testing all newborns TRECs and KRECs (15, 18). Thus, SCID was belatedly diagnosed and, as BCG vaccination is mandatory in Brazil and recommended at birth, the patient received the vaccine at four months of age, which led to BCG-itis requiring treatment. One year after the patient’s HSCT, SARS-CoV-2 was detected in nasopharyngeal samples, in addition to duodenum and intestine samples, with persistently positive results throughout a period of six months. Despite the overall low donor chimerism following HSCT, the non-critical SARS-CoV-2 infection, along with successful management of various bacterial infections, can be attributed to the residual functional *JAK3* activity derived from the transplanted cells of the patient’s father. The patient’s compromised immune response, however, was not effective enough to promote timely viral clearance, leading to the 6-month-long SARS-CoV-2 infection as well as several ICU admissions due to other bacterial infections. The patient was infected with the Gamma lineage, and samples collected and tested during the hospitalization presented the same lineage (9), strongly suggesting that the persistently positive results in SARS-CoV-2 detection tests was due to chronic persistent infection rather than any reinfection events.

*JAK3* is a member of the JAK family of tyrosine kinase proteins involved in cytokine receptor-mediated intracellular signal transduction through the JAK/STAT signaling pathway. Due to the essential role of *JAK3*- γc receptor interaction and signaling, functional variants in both *JAK3* and *IL2RG* genes can lead to autosomal recessive and X-linked recessive T-B+NK- SCID, respectively, as result of the impairment on signalization of cytokines involved in the development of B cells (IL-2, IL-4 and IL-9) and differentiation and activation of T and NK cells (IL-7, IL-15 and IL-21) (2, 19, 20). Protein structure and mutation modeling of *JAK3* indicated that the p.Leu401Pro substitution increases structural instability of the protein region, changing both the distances and the pattern of interaction with neighbor residues. The Leu401 residue is located in a highly conserved position within the SH2 domain on different species, a region that, together with the four-point-one, ezrin, radixin, moesin (FERM) domain, form a single receptor binding module known to mediate JAK association with different cytokine receptors containing the common γc. The JAK interaction with common γc receptors is mediated by a membrane-proximal site in the intracellular portion of the receptor, a region composed by the proline-rich “Box1” and the downstream hydrophobic “Box2” regions, separated by an “interbox” segment (19, 21). Substitutions in residues of both the receptor binding module and in the Box1 or Box2 regions of the intracellular portion of the γc receptor, may disrupt the JAK interaction with the cytokine receptor γc and therefore impair the cytokine signaling pathway, leading to SCID. One study evaluating EBV-transformed B (EBV-B) cells from a SCID patient observed that the p.Tyr100Cys substitution, located in the hydrophobic core of *JAK3* FERM domain, was found to disrupt the protein’s interaction with γc receptor chain, as tested by co-immunoprecipitation, and to cause constitutive *JAK3* phosphorylation (22).

Other missense variants in *JAK3* were reported affecting the same domain, such as p.Arg402His and p.Arg403Cys variants, associated with SCID (23, 24). A different study identified the p.Phe292Val variant, also in the FERM domain, that leads to an impairment of *JAK3* expression in the patient’s EBV-B cells and causes SCID in an Italian patient (25). Taken together, this evidence indicates the importance of the amino terminal FERM-SH2 region in the *JAK3* interaction with the γc receptor and point towards the pathogenic effect of different variants found in this region causing SCID. We therefore hypothesize that the variant identified in this current report patient (p.Leu401Pro) leads to greater flexibility of the SH2 domain, potentially compromising the interaction between *JAK3* FERM-SH2 region and the intracellular portion of common γc receptors, finally leading to the observed SCID phenotype. Although we were unable to perform *in vitro* functional validation of the variant, we provided compelling evidence for the reclassification of pathogenicity of the identified variant using up-to-date ACMG-AMP criteria for germline sequence variant classification (12). Two lines of pathogenic supporting evidence (PP3+ PP4+) and one line of pathogenic moderate evidence (PM2_sup), are related to the computational analysis pointing towards a deleterious effect of the variant, the fact that the patient’s phenotype is highly specific to the *JAK3* gene, and due to the absence of the identified variant in population databases, respectively. The possible fourth evidence for pathogenicity classification, also moderate, refers to PM1_sup (non-truncating non-synonymous variant located in a mutational hot spot and/or critical and well-established functional domain), as the relevance of FERM and SH2 domains to *JAK3* interaction with the intracellular tail of the γc receptors and hence downstream pathway signaling was already evidenced in previous studies. Other limitations in our study include the absence of evaluation in terms of *JAK3* expression or STAT5 phosphorylation data before and after the HSCT, as the hospital does not perform such analysis in health care assistance. We only used already stored samples for this report.

## Conclusions

This report described a novel *JAK3* missense variant (c.1202T>C) causing p.Leu401Pro substitution in the protein’s SH2 domain leading to T-B+NK- SCID in a six-months-old boy, who suffered from different infectious diseases, including BCG-itis, *P. jirovecii* pneumonia, and a prolonged SARS-CoV-2 infection, lasting for six months. This study highlights the critical importance of the implementation of newborn screening programs in order to detect SCID as well as other T and B cell deficiencies, especially in children from families with no history of immunodeficiency, avoiding the inoculation of live-attenuated vaccines, in particular in the numerous countries where vaccines like BCG are mandatory in infancy. The early detection of the disease through newborn screening also prompts the timely HSCT deployment before infections onset. In addition, performing SCID molecular diagnosis and identifying the causal genetic variant can drive donor selection strategies, factors that ultimately improve overall survival and prognosis after HSCT, besides to allow better characterization of causal mutations and genes in IEIs phenotypes and aiding the understanding of the function of non-well functionally characterized protein domains. This study provided evidence for the reclassification of pathogenicity for the novel variant identified, currently a variant of unknown significance by the ACMG criteria. Further investigations are required to better understand the molecular mechanisms of the interaction between the FERM-SH2 region of *JAK3* protein with cytokine receptor’s γc, as well as the impact of the p.Leu401Pro and other similar substitutions on its function, receptor binding, and relation to SCID pathogenesis.

## Data Availability

The original contributions presented in the study are included in the article/supplementary materials, further inquiries can be directed to the corresponding author/s.
